# Design and Implementation of Real-Time Vehicular Camera for Driver Assistance and Traffic Congestion Estimation

**DOI:** 10.3390/s150820204

**Published:** 2015-08-18

**Authors:** Sanghyun Son, Yunju Baek

**Affiliations:** School of Computer Science and Engineering, Pusan National University, Busan 609735, Korea; E-Mail: sonsang@eslab.re.kr

**Keywords:** computer vision, vehicle recognition, road traffic, vehicular network, real-time traffic estimation

## Abstract

As society has developed, the number of vehicles has increased and road conditions have become complicated, increasing the risk of crashes. Therefore, a service that provides safe vehicle control and various types of information to the driver is urgently needed. In this study, we designed and implemented a real-time traffic information system and a smart camera device for smart driver assistance systems. We selected a commercial device for the smart driver assistance systems, and applied a computer vision algorithm to perform image recognition. For application to the dynamic region of interest, dynamic frame skip methods were implemented to perform parallel processing in order to enable real-time operation. In addition, we designed and implemented a model to estimate congestion by analyzing traffic information. The performance of the proposed method was evaluated using images of a real road environment. We found that the processing time improved by 15.4 times when all the proposed methods were applied in the application. Further, we found experimentally that there was little or no change in the recognition accuracy when the proposed method was applied. Using the traffic congestion estimation model, we also found that the average error rate of the proposed model was 5.3%.

## 1. Introduction

Owing to the development of semiconductor and mobile communication technology, smart vehicular technologies that enable safety management and provide certain information to a driver using high-performance sensors and wireless communication devices in a heavy-traffic environment is an active area of research [1]. The number of vehicles has increased and road conditions have become complicated, increasing the risk of crashes [2]. Therefore, a system that provides safe vehicle control and various types of information to the driver is urgently needed.

Conventional vehicular camera systems, which record vehicle driving information in order to reveal the causes of crashes, and the advanced driver assistance system (ADAS), which facilitates driver comfort and vehicular safety, are typical examples. Further, smartphone technologies have evolved rapidly, e.g., the fast-paced, low-cost technologies associated with the development of embedded processors, image sensors, and global positioning system (GPS) sensors. As the development of these small devices is facilitated, many laboratories and vehicular navigation companies are actively pursuing the development of a conventional vehicular camera system. In addition, the study of ADASs [3,4] using various sensors and image information is stepping up research and development owing to the high performance of the various sensors considered thus far.

Although ADASs were initially studied in terms of driver convenience, these systems have recently been improved to provide safety services, such as alerts regarding lane departure and direct vehicular control. The German automotive powerhouses and relevant support agencies, such as the Technical Monitoring Association, Automobile Association, and Automobile Manufacturers Association, have been studying the effects of a variety of ADASs.

Many researchers have studied security services, such as traffic sign recognition, lane keeping, parking aids, and emergency braking. Industrialized countries have already performed the duties of such safety-related systems. An ADAS recognizes a vehicle’s surroundings using various sensors. However, ADASs have problems with low accuracy and limited types of measurable objects.

An ADAS is equipped with various radars and IR sensors to recognize various situations. There is increasing need for an image recognition sensor that can collect and utilize information about specific road conditions by using the image data recorded by a camera to maximize the utilization of the limited sensor information. Thus, research using computer vision techniques, such as pedestrian recognition and license plate and traffic sign recognition, is in progress. For example, Mobileye ADAS products [5] in Israel are equipped with system-on-chip hardware for image recognition. It is possible to determine the surrounding information by collecting image data from cameras attached to the front and rear of a vehicle. In addition, IT companies, such as Google and Apple, are performing on-going automatic driving technology research and development, and automobile manufacturing companies, such as Bayerishe Motoren Werke (BMW), Ford, General Motors (GM), Toyota, and Volkswagen (VW) are performing on-going research on ADAS technology.

For infrastructure-to-vehicle and vehicle-to-vehicle communication, researchers are developing technology for exchanging information by interradio communication. Among the in-vehicle network technologies currently in use, dedicated short-range communication (DSRC) is preferred in Korea. DSRC has a problem in that a connection with an external network cannot be established. Utilization of mobile communication networks is limited because of the associated fees. A related study based on wireless sensor networks or *ad hoc* networks is in progress [6], and the related standards for wireless access in a vehicular environment [7,8,9] are being established by the IEEE. However, it is difficult to apply an *ad-hoc*-network-based technology because of the absence of commercial IEEE 802.11p products and poor communication performance with respect to the degree of data dissemination by the device.

Traffic management technologies include intelligent transportation systems (ITSs) and transport protocol expert groups (TPEGs) [10]. ITSs will improve the efficiency and safety of traffic control by applying state-of-the-art technologies, such as electronic control and communication, to on-going traffic. Examples of ITSs that can be used in the real world include bus arrival information systems, automatic intersection signals, and real-time traffic information services provided by navigation systems. In Korea, TPEGs are used mainly for providing real-time traffic information to a vehicle navigation system. A TPEG is a protocol that provides real-time traffic and travel information through digital multimedia broadcasting. Conventional traffic management techniques determine the traffic conditions using closed-circuit televisions installed along the roads for the collection of traffic information, or by measuring the time required for a vehicle to pass a specific point. Conventional collection methods have a problem in that information collection is available only in limited areas.

A driver assistance system collects sensor data using the vehicle’s sensors. This system provides the driver with information on the area around the vehicle and uses road information for vehicle control. However, the sensors collect only limited types of road information. To address this limitation, the proposed device can collect various types of information to analyze camera images. In addition, the collected road information is available for use only to the collecting vehicle. Thus, a real-time traffic information system is needed that can gather road information from vehicles and provide traffic information to other vehicles using a mobile cellular network.

In this paper, we designed a smart driver assistance system based on image recognition and a mobile network, and we implemented the system to evaluate its performance. In addition, we designed and implemented a real-time traffic information system. We defined the smart driver assistance system and proposed methods of overcoming low image recognition throughput. In addition, we proposed a traffic analysis model that uses the collected road information from a vehicle’s sensor data. We performed several experiments to evaluate the image recognition throughput and traffic estimation performance. 

## 2. Smart Vehicular Camera

With the rapid evolution of mobile platform technology, the components of mobile devices, such as embedded processors, image sensors, gyro sensors, and GPS modules, are becoming cheaper. The proposed smart driver assistance system consists of a smart vehicular camera device and a real-time traffic information system. First, we developed a smart vehicular camera platform. The proposed platform requires high-speed camera interfaces to forward images to a processor, network interfaces to communicate with the traffic service server, and parallel processing capability to recognize input images.

### 2.1. Hardware Design 

To select an embedded processor for the smart camera platform, we performed an image recognition test using development boards. We tested four development boards, including the Samsung Exynos core and the NVIDIA Tegra core. Table 1 shows the detailed specifications of each of these four development boards. 

**Table 1:** Comparison of development board specifications.

	Arndale Exynos 5	ODROID-X2	ODROID-XU	JETSON
**CPU**	Exynos 5420 Octa Cortex™-A15 1.7 GHz quad, Cortex™-A7 quad	Exynos 4412 Quad Cortex™-A9 1.7 GHz quad	Exynos 5410 Octa Cortex™-A15 1.6 GHz quad, Cortex™-A7 quad	Tegra Kepler1 4-plus-1 Cortex™-A15 2.3 GHz
**GPU**	Mali-T628	Mali-400 MP40	PowerVR SGX544MP3	Kepler GPU with 192 CUDA
**RAM**	1 GB LPDDR3	2 GB LPDDR2	2 GB LPDDR3	2 GB LPDDR3
**Storage**	SDMMC4	SDMMC4	SDMMC4	16 GB fast eMMC4
**Power**	5 V/4 A DC	5 V/2 A DC	5 V/4 A DC	12 V/5 A DC

We evaluated the image processing performance of the development boards using the pedestrian recognition open computer vision (OpenCV) library [11] in the Ubuntu Linux environment. The input video stream for the test is a real road movie recorded using a conventional vehicular camera attached to the test vehicle. The pedestrian recognition parameters are given in Table 2.

**Table 2:** Pedestrian recognition parameters.

Parameter	Value
Library (algorithm)	OpenCV 2.4.9 (HOG)
Input image size	WVGA (800 × 480)
Size of detection windows	48 × 96
Scale	1.05
Level	13
Hit threshold	1.4
Stride	8 pixels

Table 3 compares the recognition processing performance of each device. A comparison of the recognition processing speed revealed the performance advantages of the NVIDIA Tegra core board. The results showed that the use of the GPU cores is more important than the performance of the CPU cores. To use the GPU cores, the image recognition system requires a parallel computing framework, such as OpenCL [12] or NVIDIA’s Compute Unified Device Architecture (CUDA) [13]. The CUDA library provides more options and functions than OpenCL [14,15]. Thus, the use of this library ensures high image processing performance.

**Figure 1:**
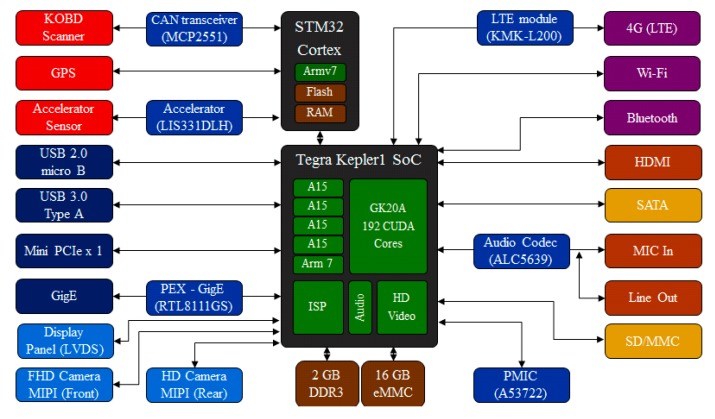
Schematic representation of smart camera platform based on NVIDIA Tegra Kepler1 core.

The proposed smart camera platform was designed using the NVIDIA Tegra Kepler1 processor and includes various modules and sensors. The platform includes a high-performance full HD camera module to collect the image frame forward of the vehicle, an LTE modem to connect to the mobile network base station, a GPS module to determine the vehicle position, and an accelerometer to determine the acceleration of the vehicle. In addition, the platform has an embedded on-board diagnostics (OBD) scanner [16,17,18] to confirm the sensor data from the vehicle interior. Figure 1 shows a schematic representation of the smart camera platform.

**Table 3:** Comparison of recognition processing performance.

Development Board	Performance (FPS)
Arndale (Exynos 5420)	1.89
Odroid-X2 (Exynos 4412)	2.21
Odroid-XU (Exynos 5410)	3.25
JETSON (NVIDIA TK1, CPU only)	3.82
JETSON (NVIDIA TK1 CPU, GPU)	15.86
PC (Intel i5)	13.82
PC (Intel i5, NVIDIA GeForce 750)	44.12

We connected a camera module and an LTE network module to an NVIDIA Jetson board [19] based on the Tegra Kepler1 core, and we designed an extension board based on the STM32 cortex core [20], which has an embedded OBD scanner, a GPS module, and an accelerometer. To read the data from the vehicle interior using sensors, we use a controller area network (CAN) transceiver for a low-layer connection and the standard OBD-II/KOBD protocols.

### 2.2. Software Design

**Figure 2:**
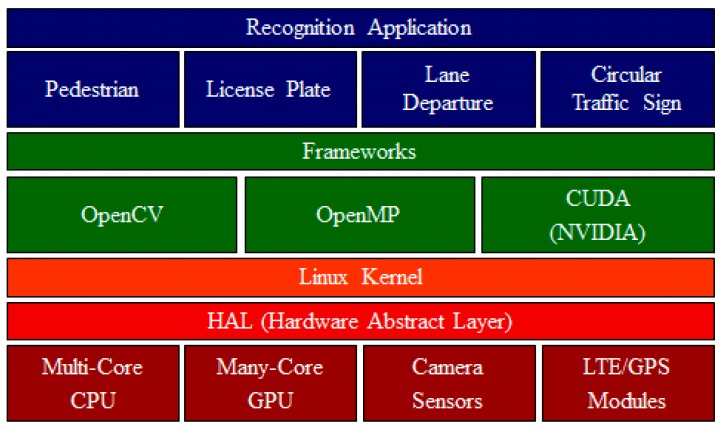
Software architecture of the proposed smart vehicular camera.

A variety of open-source software was applied to operate the smart vehicular camera device. Performing device control using the Linux kernel, we developed the recognition application using the computer vision library and implemented the application using the CPU and GPU parallel processing library [21,22,23]. The software architecture of the proposed smart vehicular camera is shown in Figure 2. The hardware abstract layer (HAL) controls various hardware devices, and the recognition application uses the OpenCV, OpenMP [24], and NVIDIA CUDA libraries to improve performance. This application uses image processing to provide road condition information, such as information about pedestrians, license plates, road lines, and traffic signs. Further, the application is designed to provide the position, speed, accelerator condition, and other vehicular status information.

The proposed smart vehicular camera uses a recognition process to search for valid targets, such as pedestrians and license plates, in the camera input image. Further, we applied a pedestrian recognition method based on the histogram of the oriented gradient (HOG) [25] in an image frame. This method calculates the changes in each pixel’s direction component and component size for the full frame of pixels. This gradient value can be used for object recognition, as it changes significantly at an object boundary, irrespective of the background. To find a pedestrian in a frame, the method uses support vector machines (SVMs) to compare the gradient value and the normalized histogram value by changing the scale levels.

The license plate recognition method finds rectangles and eliminates rectangles corresponding to non-plate objects from the rectangle list. The rectangle finding method uses pyramid blurring to remove the noise in the image, Canny edge detection [26] to find a rectangle edge, and the Dilate algorithm to sharpen the detected edge. Finally, the method calculates the contours relative to the edges and extracts rectangles from the calculated contours. The license plate recognition method finds the plate by checking for a specific pattern aspect ratio. The specific pattern consists of repeated black letters and white spaces. In addition, the proposed recognition application estimates the distance to the vehicle ahead using the pixel distance difference between a license plate and a hood. 

Lane and traffic sign recognition is performed using a Hough transform to find straight lines and circles. In the image frame, the recognition method identifies a straight line that contains most of the overlapping points [27]. Furthermore, to identify circles, the method finds the intersections of the straight lines, and these points are considered the centers of circles. The circular traffic sign recognition method performs character recognition to find circles, and the lane departure warning method determines lane departure on the basis of the inclination of the found straight line.

Image recognition techniques require high computational power and considerable processing time. Processing time is accumulated whenever each recognition method is performed. These features make it difficult to perform real-time image processing. Therefore, image recognition techniques require two methods of real-time image processing, namely, a dynamic region of interest (D-ROI) method to reduce the search area and a dynamic frame skip (DFS) method to discard unnecessary frames.

## 3. Methods of Minimizing Computing Load

In the limited environment of a mobile embedded system, we propose to use the D-ROI method to perform image recognition smoothly. An ROI is a region of interest that is searched by the image recognition process. A minimized ROI can improve the processing speed by reducing the search region. The ROI is generally set to a fixed small size to eliminate unnecessary operations in order to reduce the processing time. To accommodate the algorithms with a high computation load, such as pedestrian recognition and license plate recognition, we shortened the processing time by applying the D-ROI method, which continuously changes the ROI size by estimating the target’s location and size in the frame.

### 3.1. D-ROI Method

In the road environment, each item of information can be recognized for each target by setting the corresponding other partial region as the ROI. Thus, each recognition method sets the upper-bound ROI size to a static ROI (S-ROI) to maintain the recognition accuracy. Here, we propose a D-ROI method to further reduce the S-ROI for the pedestrian and license plate recognition methods. The lane recognition and circular sign recognition methods impose a relatively low computational load, and reducing the search region by more than the S-ROI size is difficult. Thus, the D-ROI method is not applied to these two recognition methods.

Before describing the D-ROI, we will define the S-ROI. For pedestrian recognition, we remove the non-pedestrian regions, such as the sky, the vehicle hood, and the narrow left and right sides of the full frame.

The same S-ROI is used for license plate recognition and pedestrian recognition. The S-ROI for the lane recognition algorithm is set up as the optimal region that includes the region from the vanishing point to the hood and both lanes. The S-ROI for lane departure recognition sets a sufficiently large region to determine the slope of the lane after lane recognition. Finally, the circular traffic sign recognition algorithm sets the S-ROI to the upper-right corner of the full frame.

**Figure 3:**
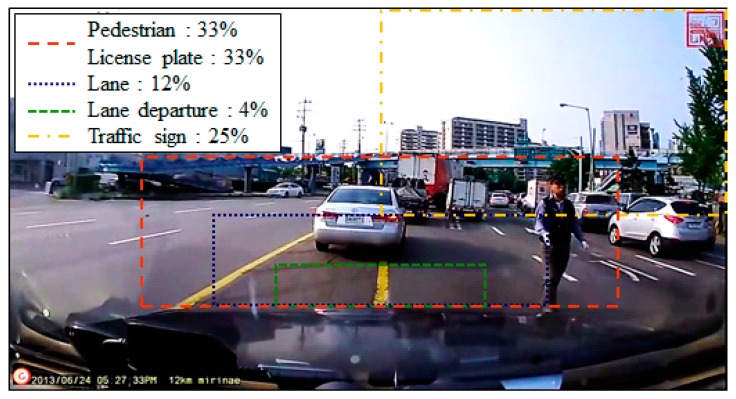
Size of static ROI for recognizing objects on the road.

This setting of the ROI also affects the recognition accuracy in addition to reducing the computational load. If similar-measurement objects appear outside of the ROI, they can be excluded, thus preventing recognition errors caused by identifying wrong targets, such as pedestrians and plates. If a camera is attached to the rear view mirror, the size of the pedestrian and license plate recognition S-ROI is 33% of the full frame size. The lane and lane departure recognition S-ROI sizes are 12% and 4%, respectively. Further, the circular traffic sign recognition S-ROI size is 25%. These S-ROI sizes are optimal values generated by the experimental data obtained from the collected real videos. Figure 3 shows the size of the S-ROI for recognizing objects on the road.

The D-ROI method produces an ROI smaller than the specified S-ROI in order to reduce the computational load. The method dynamically changes the ROI size according to the target recognized in the previous frame. If the recognition target is not present in the current frame, the recognition algorithm searches the S-ROI of the frame.

Whenever the algorithm applied for the D-ROI method recognizes a target object in the current frame, it generates a smaller D-ROI by considering a cropped image including the recognized target and the vehicle movement. The new ROI is determined by adding the movement region (MR) to the cropped region (CR). The movement region is calculated as:

(1) $${MR}_{ped}=\alpha \cdot F_{pixel}\left(({\vec{V}}_{car}+{\vec{V}}_{ped})\cdot \frac{{FR}_{skip}+1}{{FR}_{inp}}\right)+C_{MP}$$

where *V_car_* denotes the velocity of the vehicle, and *V_ped_* represents the velocity of the recognition target. The two velocity vectors are substantially orthogonal and are used to calculate the moving distance in order to estimate the MR size. Further, *FR_inp_* denotes the number of frames in the input image, and *FR_skip_* represents the value of frame skips per second. If the algorithm considers the entire frame for the calculation, this value is zero. Otherwise, if the algorithm skips frames, *FR_skip_* increases. *C_MP_* denotes the pixel margin recommended for image recognition. We set this variable at a fixed value for each algorithm and image size. Further, *α* denotes a constant value to determine *MR*. For pedestrian and license plate recognition, the D-ROI method can reduce the calculation domain on both sides.

**Figure 4:**
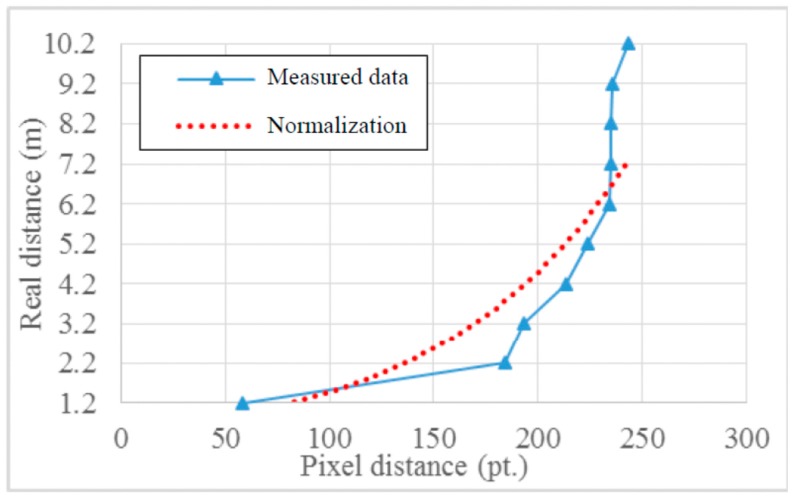
Function for converting pixel distance to real distance.

To convert real distances to pixel distances, the following equations are used:

(2) $$\begin{array}{l} F_{pixel}\left(d\right)=0.4799\cdot e^{0.0112d}\\ F_{dist}\left(p\right)=2.0837\cdot \text{ln}p-9.3599 \end{array}$$

where *F_pixel_*(*d*) denotes a function that converts the real distance to the pixel distance in the image, and *F_dist_*(*p*) denotes an inverse function of *F_pixel_*(*d*); we derived Equation (2) on the basis of the measured values. The graph in Figure 4 shows how the pixel variation corresponds to the variation in the real distance.

Considering the distance between the license plate of the vehicle in front and the hood, the D-ROI method generates the D-ROI using the following equation:

(3) $$\begin{array}{l} {MR}_{plate.}height=\alpha \cdot \left|d-d_{prev}\right|+C_{MP}\\ {MR}_{plate.}width=\beta \cdot CR_{plate} \end{array}$$

where *MR_plate_* denotes the movement region of the license plate for image recognition. The height of the ROI is set on the basis of the difference in the distance from the vehicle in front in the previous frame, and the width of the ROI is set to *β* times the width of a plate considering the extra width moveable to both sides. This D-ROI is changed continuously according to the recognition result, and the D-ROI method is executed continuously up to n times. After being executed *n* times or failing in the target search, the recognition algorithm searches for the S-ROI again to prepare for the emergence of a new target.

Whenever pedestrians or license plates are recognized in a frame, the D-ROI method reduces the computational load significantly. However, the D-ROI method has a problem in that it is not applied when vehicles or pedestrians do not appear in front of the vehicle. When a frame is processed every moment, there is no problem in the precision or accuracy; however, it is difficult to save computing resources. For example, when a vehicle is driven at a constant speed on the highway, the change in the image is small. When the speed of the vehicle is very slow, there is little change in the continuous input image. In such cases, the recognition algorithm can sufficiently detect the situation by low frequency image processing. 

### 3.2. DFS Method

Here, we propose the DFS method to solve this problem. The proposed method needs certain parameters for discarding frames, such as the vehicle’s speed and acceleration variation values. If the vehicle’s speed is fast, the image changes more frequently; thus, the skip rate is set to a low value. In contrast, if the rate of change is small at a low speed, the skip rate can be increased. The acceleration variation value affects acceleration, deceleration, impacts, and turning. When the change in the acceleration value is large, the image changes suddenly; thus, the skip rate is set to a low value. The speed and acceleration variation values are interdependent; therefore, we designed the model for frame skip rating by using these two parameters.

This model is expressed as:

(4) $$FR_{skip}=\text{min}\left(\alpha \cdot \left(\frac{s-\text{min}(s)}{\text{max}(s)-\text{min}(s)}\right)\cdot \left(1-\frac{\Delta A-\text{min}(\Delta A)}{\text{max}(\Delta A)-\text{min}(\Delta A)}\right),FR_{inp}\right)$$

After standardizing the two values, we derived an equation to assign a weight to each value. Here, max(s) and min(s) are based on the speed limits of the road, and max (∆A) and min (∆A) are set to the average of the acceleration variation values generated according to the typical behavior of the driver. Algorithm 1 presents the pseudocode of the D-ROI and DFS methods for minimizing the computing load.

**Algorithm 1.** Pseudocode of D-ROI and DFS methods for minimizing computing load.*frame* ← the frames from camera device *s* ← vehicular speed value Δ*A* ← vehicular acceleration value **Initialised**
*ROI* = S-ROI, *frame_count* = 0, *D-ROI_count* = 0, *target_searching* = false **for all**
*frame*
**do**    *frame _count* = (*frame _count* + 1) % *FR_inp_*    *FR_skip_*% = min [α × standardisation(*s*) × standardisation (Δ*A*), *FR_inp_*]    **if**
*FR_skip_* % *frame_count* = 0 **then**        **if**
*ROI* ≠ S-ROI **then**           *D-ROI_ count* += 1        **end if**    **else then**        *target_searching, CR* = recognition_algorithm (*ROI*)        **If**
*target_searching* = false **or**
*D-ROI_ count* > *D-ROI_* threshold **then**           *ROI* = S-ROI           *D-ROI_ count* = 0        **else then**           *D-ROI* = *CR* + *MR*           *ROI* = *D-ROI*           *D-ROI_ count * += 1        **end if**    **end if** **end for**

## 4. Real-Time Traffic Information System

Traffic information can be categorized as incident and traffic flow information. Examples of incident information are information on crashes, road construction, and road restrictions. Incident information can be easily obtained by having the parties involved send the information to the main server. However, it is difficult to estimate traffic flow information using a single data item from a vehicle, as computing the time at which the vehicle passes along a specific road is difficult. Therefore, we propose a method of estimating traffic congestion using the vehicle-to-vehicle distance, vehicle speed, and number of neighboring vehicles provided by the proposed smart vehicular camera. The number of neighboring vehicles is difficult to provide for each vehicle using the collected position information of each vehicle.

The proposed model for estimating the traffic congestion degree is as follows: The server divides the road into several sections and then initializes the value of the number of neighboring vehicles for each section. Whenever the server receives vehicle data, it checks the position information to find the section and updates the value of the number of neighboring vehicles. We designed the model to use a combination of vehicle-to-vehicle distance, vehicle speed, and number of neighboring vehicles. In this case, we need a proper scoring function to use each data item and apply the linear combination for normalization. The proposed model for estimating the traffic congestion degree *C_traffic_* is expressed as:

(5) $$C_{traffic}=w_{s}S(x)+w_{n}N(y)+w_{d}D(z)$$

Here, *w_s_* represents the speed weight value, *w_n_* indicates the neighboring vehicle’s weight value, and *w_d_* denotes the intervehicle distance weight value. *S*(*x*), *N*(*y*), and *D*(*z*) denote the scoring functions for converting the speed, neighboring vehicle, and intervehicle distance values, respectively. To validate the model, we designed simple scoring functions intuitively. A road generally becomes congested when the vehicle speeds are slow, there are many vehicles on the road, or the intervehicle distance is small. Considering these features, we designed the simple scoring functions expressed in Equations (6)–(8).

(6) $$S_{simple}(x)=1-0.005x$$

(7) $$N_{simple}(y)=0.05y$$

(8) $$\begin{array}{l} D_{simple}(z)=1-0.035z,(0<z<20)\\ D_{simple}(z)=0.3-0.00375(z-20),(20\leq z) \end{array}$$

The ground truth (GT) value of a road is calculated to represent the ratio of the estimated time of passing along the road at limited full speed to the actual time required to pass along the road. The GT value of the congestion degree is given by:

(9) $$GT=\frac{AT_{measured}}{S_{lmt}/Dist_{road}}$$

Here, *AT_measured_* denotes the measured actual time along the road, *S_lmt_* represents the limited maximum speed on the road, and *Dist_road_* indicates the length of the road.

Comparing the GT of traffic congestion and the estimated congestion degree using the simple scoring functions, we found that the trend of the two graphs was similar, but the measurement error was 49%. To minimize this error, we thoroughly analyzed the congestion situation and designed the following improved scoring functions.

(10) $$S_{improved}(x)=5x^{-1}+C_{s}$$

(11) $$N_{improved}(y)=0.125{C_{n}}^{y}$$

(12) $$D_{improved}(z)=\sqrt{1-10^{-4}z^{2}}$$

To obtain these improved functions, we first analyzed the traffic congestion according to the vehicle speed. The vehicle speed was slow when the road was congested and fast when the road was empty. However, when the speed exceeded the threshold speed, the speed depended on the nature of the driver rather than the congestion degree. Therefore, we designed an improved speed scoring function that is sensitive to the speed change in the low-speed range. Second, we analyzed the traffic congestion according to the intervehicle distance; below a threshold, the intervehicle distance depended on the nature of the driver. Thus, the distance affected the congestion degree only slightly. In addition, if the distance exceeded a threshold, it was difficult to determine whether the distance was valid information. Therefore, we designed the intervehicle scoring model of elliptic functions to be sensitive to changes in the intermediate range. Third, we analyzed the traffic congestion according to the neighboring vehicles on the road. The number of neighboring vehicles did not significantly affect the congestion degree when it was less than the capacity of the road. In contrast, if the capacity of the road was exceeded, the congestion degree increased rapidly. Therefore, we designed the improved scoring model having an exponential form to reflect this tendency.

## 5. Performance Evaluation

We implemented the smart driver assistance system before evaluating its performance.

### 5.1. Implementation for Performance Evaluation 

**Figure 5:**
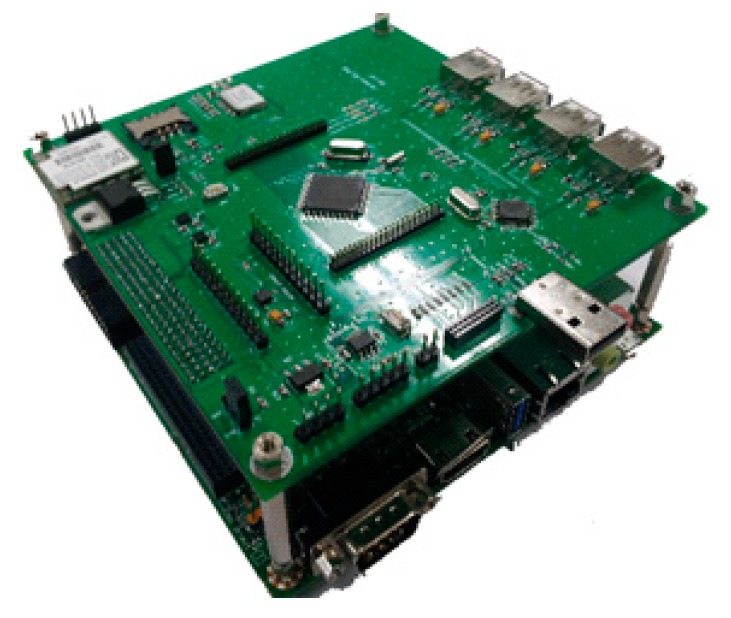
Prototype of the smart vehicular camera device based on NVIDIA Jetson board with the proposed extension board.

To implement the smart vehicular camera device, we used a high-performance application processor for image processing and a coprocessor for sensor data collection. In this study, we selected NVIDIA’s Tegra Kepler1 AP and STM32 cortex microprocessors. To configure the smart camera device, we used the NVIDIA Jetson development board based on the Tegra Kepler1 core and added an expansion board having the STM32 core and sensors. Figure 5 shows the prototype of the smart vehicular camera.

The extension board included the accelerator sensor module, GPS module, and CAN transceiver. The GPS module provided the position information of the vehicle via the UART interface, and the accelerometer, along with an analogue-to-digital converter, provided the acceleration data of the vehicle. In addition, the CAN transceiver received commands from the extension board and read the sensor data of the vehicle interior using the OBD-II protocol. The hardware specifications of the smart vehicular camera are given in Table 4.

To make the best use of the resources of the AP for high-speed image processing, we implemented the image recognition algorithm using parallel processing by the multicore CPU and many-core GPU. We implemented task parallelism considering the Tegra Kepler1 processor, which contained four CPU cores.

Task parallelism is generally classified as functional separation or data separation. In functional separation, tasks are mapped according to the function of the program executed in parallel. Data separation refers to the mapping of different tasks according to the data required to perform the same operation. To improve the performance of the image recognition algorithms with respect to functional separation of the four recognition processes, we implemented a parallel processing program.

**Table 4:** Hardware specifications of smart vehicular camera.

Mainboard	NVIDIA Jetson Tegra Kepler1
Microprocessor	STM32F105
LTE module	KMK-L200
GPS module	GMMU1
Accelerator sensor	LIS331DLH
CAN transceiver	MCP2551
USB hub controller	TUSB2046
Board size	127 mm × 127 mm × 35 mm

We implemented a recognition algorithm that required the operating system to have the scheduling threads in parallel, assigning threads to each main function by applying the open-source library of OpenMP. To improve the processing performance, detailed functions, such as pedestrian, license plate, lane departure, and circular sign recognition were assigned to each of the GPU cores. 

To perform license plate recognition, we allocated parts of the pyramid operation, such as image upscaling and downscaling, to the GPUs. In particular, we allocated the interpolation and extrapolation processes for performing the pyramid operation to the GPU cores, and the operating result was obtained through a shared memory between the CPU and the GPU. Similarly, we allocated Sobel filtering, obtaining the magnitude of a vector for Canny edge detection, and calculating the hysteresis for the gradient classification to the GPU cores.

In pedestrian recognition, the functions dispersed to the GPU cores were the oriented gradient calculation, histogram calculation, and SVM classifier. In lane departure and circular sign recognition, the Hough transform to extract straight lines and circles was allocated to the GPU cores.

Thus, the functions were implemented with CUDA and parallel-processed by the GPU cores to improve the performance. In addition, we set the maximum number of threads considering the characteristics of the smart vehicular camera and assigned the threads to the tasks. We assigned 128 threads, which did not exceed the 192 GPU cores of the NVIDIA Tegra Kepler1, and thus minimized the waiting threads, which occur when the number of threads exceeds the number of GPU cores. The optimized program was implemented on the smart vehicular camera device.

A prototype of a traffic information server was implemented to evaluate the performance of the estimation of the traffic congestion degree. A server for collecting the information from vehicles sent through a mobile communication network was implemented; it executed a function to estimate the congestion degree of roads using the collected information.

### 5.2. Image Processing in the Vehicle Environment

**Figure 6:**
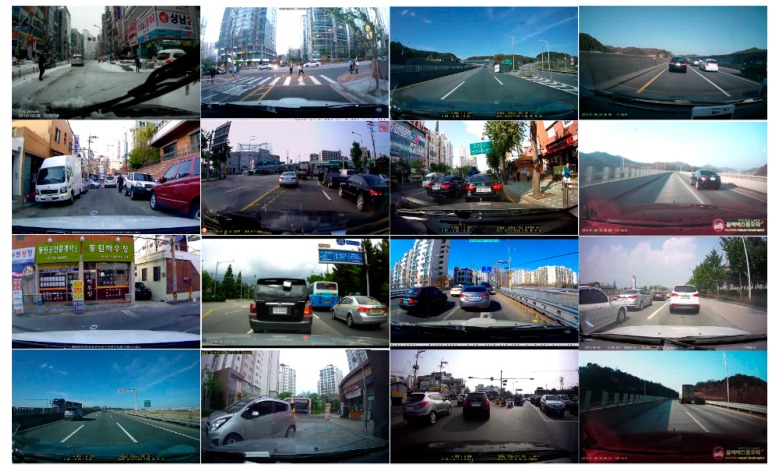
Twelve sample images recorded by a conventional vehicular camera and used for the performance evaluation (24 fps, MPEG-4, WVGA, and 10 s).

The performance of the implemented smart vehicular camera was tested using the recorded driving image sets. The 16 sample images were recorded using a conventional vehicular camera with a frame rate of 24 fps, MPEG-4 compression, and WVGA resolution (800 × 480), and the length of each video was 10 s. In other words, we evaluated the computation time and accuracy using 3840 frames. Figure 6 shows frames from the recorded driving image sets. 

We evaluated the processing power using the D-ROI method, DFS method, and parallel processing implemented on the smart vehicle camera device. The ROI technique was used to evaluate the pedestrian recognition and license plate recognition performance, and the DFS method and parallel processing were evaluated for four-image recognition. The evaluation parameters were the image processing time and accuracy. The image processing time was evaluated according to the availability of real-time operations based on the cumulative run time, and the accuracy was evaluated on the basis of whole image processing with respect to the ROI size change and number of frame skip changes. 

**Figure 7:**
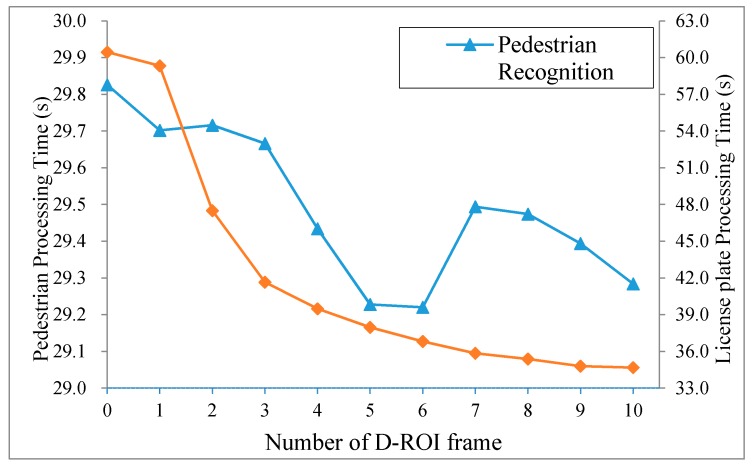
Recognition processing time for the number of D-ROI frame.

First, we conducted a performance evaluation to select the optimal value of *n* in the D-ROI method for each of the five sample images (pedestrian and license plate). Figure 7 shows the recognition processing time according to the number of D-ROI frames. Table 5 shows the accuracy of the pedestrian and license plate recognition algorithms according to the number of D-ROI frames. Increase in *n* results in the reduction of the processing time and accuracy. We have confirmed that the accuracy suffers because the recognition algorithms often do not check the entire region when *n* increases. It is difficult to determine the values, since the optimal value *n* depends on the conditions, such as the power level of the processor, complexity of the algorithm, and quality of the input image. Therefore, we used an optimal value based on the average of evaluation results, and other experiments were performed. 

**Table 5:** Comparison of recognition accuracy for number of D-ROI frame *n.*

*n*	Pedestrian Recognition					License Plate Recognition				
	image#1	image#2	image#3	image#4	image#5	image#6	image#7	image#8	image#9	image#10
**0**	95.28%	31.52%	95.00%	54.55%	43.66%	99.25%	77.08%	11.62%	59.49%	98.67%
**1**	87.74%	29.35%	81.67%	45.45%	39.44%	99.25%	77.08%	11.62%	59.49%	98.67%
**2**	87.74%	27.17%	86.67%	50.00%	40.85%	99.63%	76.67%	11.62%	59.49%	98.67%
**3**	79.25%	27.17%	76.67%	36.36%	45.07%	99.25%	77.08%	11.62%	59.24%	98.67%
**4**	83.96%	26.09%	81.67%	45.45%	39.44%	99.63%	77.08%	11.62%	59.24%	98.67%
**5**	85.85%	26.09%	80.00%	47.73%	40.85%	99.63%	77.08%	11.62%	59.24%	98.67%
**6**	85.85%	28.26%	75.00%	40.91%	42.25%	99.63%	77.08%	11.62%	59.24%	98.67%
**7**	81.13%	26.09%	81.67%	45.45%	43.66%	99.63%	77.08%	11.62%	59.24%	98.67%
**8**	81.13%	26.09%	81.67%	36.36%	40.85%	99.63%	77.08%	11.62%	59.24%	98.67%
**9**	87.74%	29.35%	88.33%	40.91%	43.66%	99.63%	77.08%	11.62%	59.24%	98.67%
**10**	83.02%	25.00%	80.00%	43.18%	42.25%	99.63%	77.08%	11.62%	59.24%	98.67%

**Figure 8:**
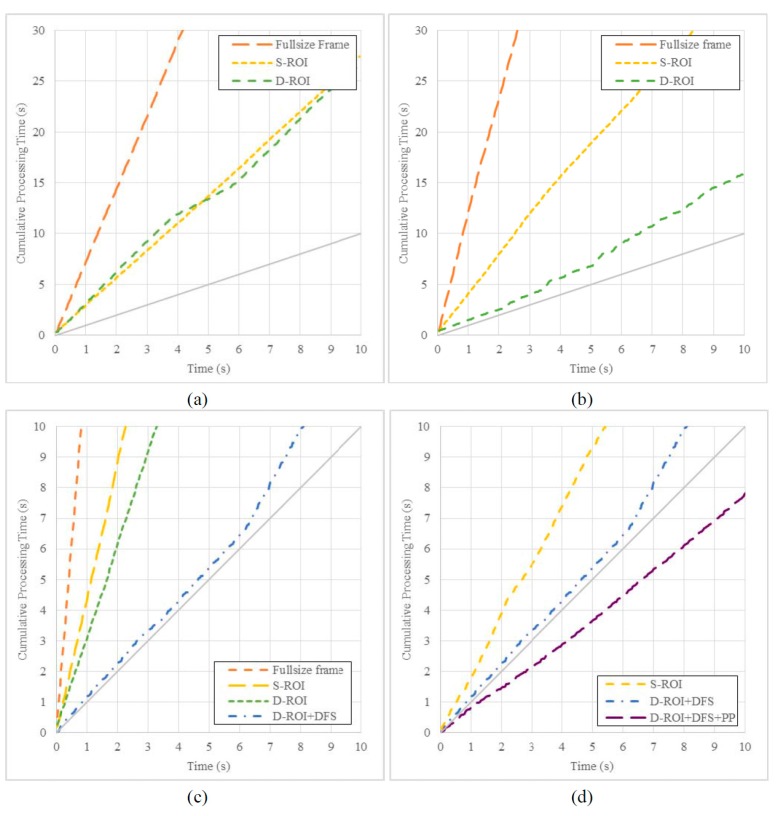
Evaluation of image processing in the vehicle environment using the proposed methods, D-ROI, DFS, and parallel processing (PP). (**a**) Pedestrian recognition; (**b**) license plate recognition, and comparisons of (**c**) computation load and (**d**) processing time for all four algorithms. (D-ROI frame n = 5).

Second, we evaluated the performance of the D-ROI method. Figure 8a,b show graphs comparing the processing time according to the ROI size. The gray solid lines in the graphs are an upper bound to verify the real time processing; thus, when image processing was performed in real time, the slope of the cumulative processing time line was lower than the slope of the solid line. 

Figure 8a shows the cumulative processing times of pedestrian recognition for several ROI sizes. Compared with the result for the full frame obtained by processing the entire input image, the results of the two considered methods show a higher throughput due to a reduced calculation region. In the intervals of 4–6 s and 9–10 s, we found that the gradient of the result of the D-ROI was lower, as the pedestrian was recognized in a previous frame, and the current processing region was reduced.

Figure 8b shows the cumulative processing time of vehicle license plate recognition with respect to the ROI size. The D-ROI method showed a faster processing time than the full frame. It was slightly faster than the S-ROI method for longer time duration. The processing time of the D-ROI was always the shortest because the license plate always appeared in the sample image, and the D-ROI method was applied consistently. Thus, the processing time of the S-ROI method was reduced compared to that of full frame processing by considering part of the full frame, and the processing speed of the D-ROI method was the fastest because the D-ROI was smaller than the S-ROI. However, it was difficult to ensure real-time processing that always processed the minimized region. 

We evaluated the processing performance of the four recognition algorithms by applying the D-ROI method, frame skip method, and parallel processing. Figure 8c,d show the results of the performance evaluation. Further, Figure 8c shows the effect of minimizing the computation load using the D-ROI and DFS methods on the image recognition algorithm. The DFS method improved the processing performance by reducing the number of frames processed.

Figure 8d compares the processing time of all the considered methods. The results reveal the shortest processing time and confirm that real-time processing is possible. In other words, we confirmed that real-time image processing could be performed using the proposed methods.

**Table 6:** Comparison of processing time for various combinations of proposed methods.

Combination of Methods	Processing Time	FPS
D-ROI + DFS + PP	7.81	30.73
D-ROI + DFS	13.43	17.87
D-ROI + PP	20.62	11.64
D-ROI + PP (GPU only)	21.29	11.27
D-ROI + PP (CPU only)	22.84	10.51
S-ROI + PP (GPU only)	36.69	6.54
D-ROI	27.04	8.88
S-ROI	42.88	5.60
Full + PP (GPU only)	93.27	2.57
Full	120.63	1.99

Table 6 shows the processing time and FPS for each of the methods and their combinations. When the image recognition algorithm was executed for a full frame of about 10 s, the image processing time was 120 s. However, when the proposed methods, D-ROI, DFS, and parallel processing, were applied, the processing time was only 7 s.

Accuracy (ACC) is the proportion of true results (both true positives and true negatives) among the total number of cases examined. Recall relates to the test’s ability to correctly detect patients who do have a condition. Precision is defined as the proportion of the true positives against all the positive results (both true positives and false positives). 

We found experimentally that there was little or no change in the recognition accuracy when the proposed methods were applied. Table 7 and Table 8 show the accuracy, recall, and recognition precision of the pedestrian and license plate recognition algorithms, respectively, for an image of about 240 frames. These results showed that the accuracy did not change significantly for any image. In the comparison of the S-ROI and D-ROI, despite the fact that narrowing of the ROI increased the accuracy of the D-ROI, the target region for recognition was reduced when the false negative rate decreased. The accuracy of the D-ROI and DFS methods increased, as these methods used information from the previous frame to save time. That is, an image recognized in the previous frame determined the next skipped frame before the consideration of a new frame for recognition. Therefore, the accuracy increased. It was useful that the change in the recognition target between frames was subtle.

**Table 7:** Comparison of recognition accuracy, recall, and precision for the proposed methods (pedestrian recognition).

	Full-Size Frame			S-ROI			D-ROI			D-ROI + DFS		
	ACC	Recall	Precision	ACC	Recall	Precision	ACC	Recall	Precision	ACC	Recall	Precision
**Image #1**	0.71	0.53	1.00	0.71	0.53	1.00	0.72	0.43	1.00	0.77	0.58	1.00
**Image #2**	0.51	0.73	0.93	0.50	0.71	0.91	0.58	1.00	1.00	0.56	0.21	1.00
**Image #3**	0.97	0.43	0.97	0.96	0.43	0.97	0.96	0.44	1.00	0.97	0.45	1.00
**Image #4**	0.92	0.57	0.81	0.91	0.57	0.81	0.94	0.23	1.00	0.93	0.38	1.00
**Image #5**	0.74	0.05	0.83	0.74	0.04	0.83	0.77	0.16	0.81	0.78	0.20	0.81
**Average**	0.77	0.46	0.91	0.76	0.46	0.90	0.79	0.45	0.96	0.80	0.36	0.96

**Table 8:** Comparison of recognition accuracy, recall, and precision for the proposed methods (license plate recognition).

	Full-size Frame			S-ROI			D-ROI			D-ROI + DFS		
	ACC	Recall	Precision	ACC	Recall	Precision	ACC	Recall	Precision	ACC	Recall	Precision
**Image #6**	0.63	0.63	1.00	0.63	0.63	1.00	0.46	0.50	1.00	1.00	1.00	1.00
**Image #7**	0.76	0.73	1.00	0.76	0.73	1.00	0.76	0.75	1.00	0.76	0.75	1.00
**Image #8**	0.09	0.09	1.00	0.09	0.09	1.00	0.07	0.09	1.00	0.14	0.17	1.00
**Image #9**	0.92	0.92	1.00	0.92	0.92	1.00	0.92	0.92	1.00	0.91	0.90	1.00
**Image #10**	0.90	0.86	1.00	0.90	0.86	1.00	0.90	0.86	1.00	0.90	0.82	1.00
**Average**	0.66	0.64	1.00	0.66	0.64	1.00	0.62	0.62	1.00	0.74	0.72	1.00

### 5.3. Estimating Traffic Congestion

We experimentally evaluated the proposed model of estimating the traffic congestion degree. When the proposed model estimated the congestion degree, we determined the estimation error by comparing the estimated results with the GT of the congestion degree according to the changes in the parameter values.

We evaluated 16 sample images recorded using a conventional vehicular camera: eight traffic congestion images and eight light traffic images. As with the conventional evaluation conditions, each image had a frame rate of 24 fps, MPEG-4 compression format, and WVGA resolution, and the length of each video was approximately 10 s. We defined the road conditions using Average Annual Daily Travel (AADT). The term *traffic count* is used to refer to an AADT, which is the annualized average 24-h volume of vehicles at a given point or section of highway. It is normally expressed as the ratio of the volume of vehicles during a given period to the number of days in that period. The light traffic condition is a vehicle count less than 50,000, and the traffic congestion condition is a vehicle count greater than 50,000.

The traffic congestion images, we used six highway images and six urban images; the speed limit on the highway was assumed to be 100 km/h, and the speed limit in the urban areas was assumed to be 60 km/h. For the light traffic images, we used only highway images. We determined the error of the estimated value by comparing the GT value and the estimated traffic congestion value. 

To determine the error rate with respect to the proposed scoring functions, the performance evaluation of the proposed traffic congestion estimation model combined simple and improved scoring functions. For estimating the congestion degree when the proposed model was used, we required the scoring functions and the weight values. In the experiment, we set the weight values at a ratio of about 2:1:1.

**Figure 9:**
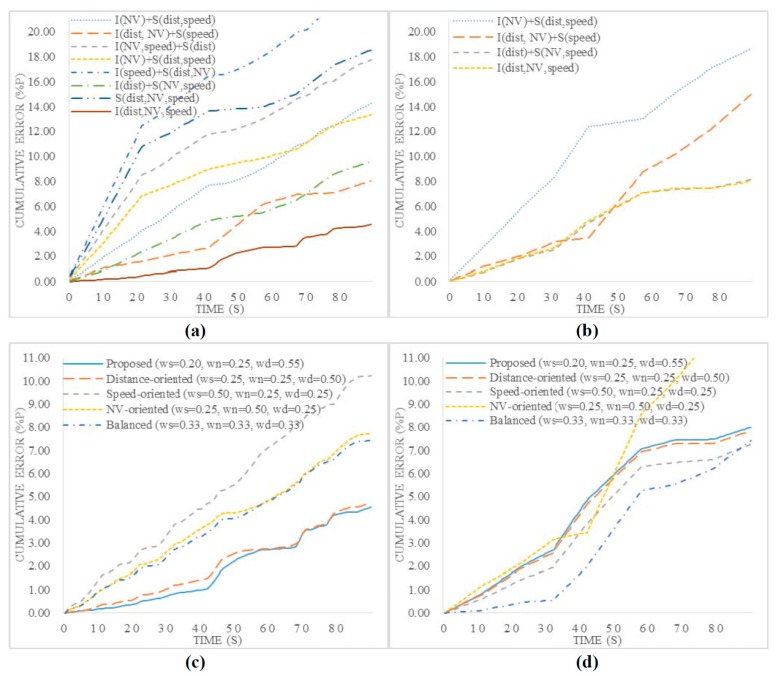
Cumulative error for various combinations of scoring functions (**a**,**b**) and weight values (**c**,**d**). ((**a**,**c**) Congested traffic, (**b**,**d**) light traffic.)

We performed experiments for a congested road and a light traffic road. For the light traffic road, it was difficult to recognize vehicles that were too far away. Therefore, only two functions, namely, the speed and neighboring vehicle scoring functions were used in the test. Figure 9a compares the cumulative error for the scoring functions for the congested road. In this figure, all the functions with improved scoring functions exhibit the best performance, as the function resolutions are set according to the features of each entry.

For the light traffic road, as shown in Figure 9b, we observed slight differences among all the results except those obtained when the improved scoring functions for speed and neighboring vehicles were applied. In contrast to the congested case, the vehicle speed was very fast, and the number of neighboring vehicles was too small. Thus, we found that the result was small irrespective of the type of scoring function. Table 9 summarizes the average error value according to the combination of the scoring function types. The experimental results demonstrated that the improved scoring functions operated correctly irrespective of the road conditions. 

Further, we performed an experiment to determine the optimal weight values for the proposed traffic congestion estimation model. In this experiment, we measured the cumulative error by changing the three weight values and road conditions. We set up various test combinations, such as a combination of the same weight values and one using an additional weight along with an existing weight value. 

Figure 9c,d compare the cumulative errors according to the combinations of weight values. For the congested road, a higher weight was set for the intervehicle distance that showed the best performance. Under this road condition, the variations in the vehicle speed and number of neighboring vehicles were small. In contrast, the intervehicle distance was highly likely to change dynamically. Thus, the estimated traffic congestion degree value was similar to the GT value when a higher weight value was applied to the intervehicle distance. In a comparison of the speed-oriented weight and neighboring-vehicle-oriented weight, the number of neighboring vehicles was a more important factor affecting road congestion than the vehicle speed.

**Table 9:** Average error rate for combinations of scoring functions under the congestion and light traffic conditions.

Combination of Scoring Functions	Congestion		Light Traffic	
	Ave.	Std.	Ave.	Std.
*I*(NV) + *S*(dist, speed)	0.158	0.056	0.207	0.105
*I*(dist, NV) + *S*(speed)	0.090	0.066	0.166	0.097
*I*(NV, speed) + *S*(dist)	0.197	0.119	0.173	0.105
*I*(NV) + *S*(dist, speed)	0.148	0.100	0.086	0.065
*I*(speed) + *S*(dist, NV)	0.262	0.184	0.207	0.105
*I*(dist) + *S*(NV, speed)	0.107	0.045	0.090	0.061
*S*(dist, NV, speed)	0.206	0.172	0.090	0.061
*I*(dist, NV, speed)	0.051	0.061	0.089	0.061

Among the experimental results obtained under the light traffic condition, that obtained using a balanced weight was the best, and that obtained using a speed-oriented weight exhibited acceptable performance. Under this road condition, the vehicle speeds changed frequently, the intervehicle distances varied considerably, and the variation in the number of neighboring vehicles was dynamic. Therefore, the use of an appropriate balanced weight for the vehicle speed and number of neighboring vehicles yielded a low cumulative error. The vehicle speed was a more important parameter for the estimation of the congestion degree, as the variation in the number of neighboring vehicles was small in the test images used in the experiment. Thus, the use of a speed-oriented weight yielded better performance than the use of a neighboring-vehicle-oriented weight.

Under the light traffic condition, the vehicle recognition success rate was low because the intervehicle distance was large. In contrast to the congested road condition, under this condition, the estimation performance deteriorated when an intervehicle-distance-oriented weight was used.

The detailed results of each experiment are shown in Table 10. On the basis of these experimental results, we confirmed that the use of a balanced weight under the light road condition and that of an intervehicle-distance-oriented weight under the congested road condition yielded good estimation performance.

**Table 10:** Average error rate for combinations of weight values under the congested and light traffic conditions.

Combination of Weight Values	Congestion		Light Traffic	
	Ave.	Std.	Ave.	Std.
Balanced	0.083	0.050	0.083	0.072
Distance-oriented	0.053	0.057	0.087	0.061
Speed-oriented	0.114	0.069	0.081	0.059
NV-oriented	0.086	0.045	0.162	0.093
Proposed	0.051	0.061	0.089	0.061

Considering both road conditions, we found that the proposed weight was similar to the intervehicle-distance-oriented weight with an approximately 5% total error rate, which was similar to the total error rate of the balanced weight. However, the traffic congestion degree should be estimated accurately under the congested road condition. Therefore, the proposed weight was found to be the most suitable for estimating the traffic congestion degree.

## 6. Related Works

### 6.1. Advanced Driver Assistance Systems

An ADAS is a vehicular device that recognizes several conditions, such as the vehicle status, driver’s condition, and environmental status to reduce the driver’s burden and enhance his/her convenience. Unlike an active safety system, which prevents a crash or reduces injury to the driver before and after a crash, an ADAS provides safety and convenience by assisting the driver during normal driving [28]. To enable such systems to provide a variety of information, many researchers are developing a technique for continuous recognition of images from a camera. 

The key functions of an ADAS are to recognize road conditions, such as pedestrians, lane departure, traffic lights, and traffic signs. The use of ADASs with these functions is likely to continue to increase because of changes in the social structure and regulatory strengthening. Consumer demand for these systems is increasing continuously, and technological developments and mass production are reducing the production cost of these systems. Therefore, the ADAS market is expected to grow by an annual average of 25% by 2017 [29].

### 6.2. Pedestrian Recognition

Of the many functions of ADASs, pedestrian recognition has been widely studied, and several algorithms for this purpose have been developed [30,31]. The widely and readily available methods include those using the object recognition method of Viola and Jones [32] and the pedestrian recognition method of Dalal and Triggs [25]. It is possible to recognize pedestrians using Haar-like features and the AdaBoost classification algorithm of Viola and Jones in a relatively small window size of 14 × 28.

The method developed by Dalal and Triggs [25] may recognize a pedestrian via an SVM classification based on the histogram of the oriented gradient (HOG). These two methods can be easily used via the OpenCV library. MULTIFTR [33] showed how a combination of Haar-like features, shapelets, shape context, and HOG features outperforms any individual feature. HOGLBP [34] combined a texture descriptor based on local binary patterns (LBP) with HOG. Fastest Pedestrian Detector in the West (FPDW) [35] was extended to fast multiscale detection. The algorithm was demonstrated how feature computed at a single scale can be used to approximate feature at nearby scales. 

FTRMINE [36] explores possibly infinite feature spaces using various strategies including steepest descent search prior to training a boosted classifier. FEATSYNTH [37] were improved by FTRMINE, the algorithm presented a scheme for combining and synthesizing a rich family of part based features. POSEINV [38] used a part-template structure to model a pedestrian parts, such as the head, body with arms and legs, and extracted HOG appearance descriptors along the local part’s outline. Table 11 shows the comparison of pedestrian recognition algorithms [39].

**Table 11:** Comparison of pedestrian recognition algorithms.

		HOG	MULTIFTR	HOGLBP	FPDW	FTRMINE	FEATSYNTH	POSEINV
Features	gradient histogram	√	√	√	√	√	√	√
	gradients				√	√		
	grayscale		√		√	√		
	color				√	√		
	texture			√			√	
Learning	classifier	Linear SVM	AdaBoost	Linear SVM	AdaBoost	AdaBoost	LinearSVM	AdaBoost
Recognition Details	window height	96	96	96	100	100	96	96
	scales	~14	~14	14	10	4	-	~18
	fps	0.239	0.072	0.062	6.492	0.080	-	0.474
	miss rate	68%	68%	68%	57%	74%	60%	86%
Implement	training data	INRIA						

In addition to these pedestrian recognition techniques, research on a fusion of several computer vision techniques and ADASs has been actively conducted [28]. Raphael *et al.* [40] proposed a method of preventing collisions using vehicle recognition, and Guo *et al.* [41] proposed road recognition techniques for conflict prevention, lane departure prevention, and cruise control.

### 6.3. License Plate Recognition

License plate recognition is important that can be recognizing neighbor vehicles and estimating vehicle-to-vehicle distance. Hongliang *et al.* [42] demonstrated a hybrid license plate extraction algorithm based on edge statistics and morphology for highway ticketing systems. This algorithm consists of the following four sections: vertical edge detection, edge statistical analysis, hierarchical-based LP location, and morphology-based LP extraction. Kim *et al.* [43] employed the vertical edges of a vehicle image, applied by image enhancement and a Sobel operator. The algorithm removes most of the background and noise edges, and searches for a license plate using a rectangular window. 

Comelli *et al.* [44] presented the RITA system for the identification of vehicular license plates. The license plate location module of the RITA system was based on the structure of the Italian license plate, which is rectangular and contains a white background with black characters. Thus, the algorithm selects the license plate area that demonstrates the maximum local contrast that corresponds to the rectangle that contains the license plate. Draghici *et al.* [45] used horizontal scanning of the image to search for the license plate location. The algorithm set the assumptions that the contrast between the background and the characters of the license plate is fine and that there are at least three or four characters on the plate. Anagnostopoulos *et al.* [46] presented an adaptive image segmentation technique of sliding concentric windows (SCW), which is considered for license plate location. The SCW method was demonstrated to describe the local irregularity in the image. The method uses image statistics values, such as the standard deviation and the mean as a heuristic, to search possible license plate location. 

In Cao *et al.* [47], the basic idea of recognition algorithm is that the color combination of a background and character is unique, and this combination occurs almost in a license plate region. In Zimic *et al.* [48], The concepts of brightness and darkness, which are demonstrated in the algorithm, are described as a fuzzy set with membership functions on the interval [0, 225], where the black represents 0, and the white represents 255 in a gray scale. In Chang *et al.* [49], their approach uses an edge detector sensitive to only three kinds of edges, black–white, green–white, and red–white, as this algorithm focuses on Korean license plates. Thus, the method generates an initial edge image in which all other color tones are eliminated. Table 12 show the comparison of license plate recognition algorithms. 

**Table 12:** Comparison of license plate recognition algorithms.

	Processing Method		Minimum Plate Size	Recognition Success Rate
Hongliang *et al.*	Binary	Edge statistics	65 × 20	99.6%
Kim *et al.*	Binary	Sobel operation	65 × 20	96.5%
Comelli *et al.*	Gray-level	Global Image Processing	100 × 25	84.2%
Draghici *et al.*	Gray-level	Global Image Processing	100 × 25	98.5%
Anagnostopoulos *et al.*	Gray-level	Region segmentation	61 × 20	87.8%
Cao *et al.*	Color	Model transformation	41 × 13	100%
Zimic *et al.*	Color	Fuzzy set theory	120 × 35	97.0%
Chang *et al.*	Color	Fuzzy set theory	80 × 45	97.6%

Further, license plate recognition and image recognition methods are now widely used. License plate recognition is used for various applications, such as illegal parking crackdown, number recognition for speeding cars, and checking out vehicles [50].

### 6.4. Intelligent Transportation Systems

An Intelligent Transportation Systems (ITS) provides traffic information and services by incorporating IT technologies into transportation facilities; further, ITSs improve the efficiency and reliability of public transport. ITSs are used in a variety of areas, including traffic management optimization, electronic payment processing, traffic information distribution activation, providing advanced traveller information, transit activation, realizing efficient freight, and providing high-tech roads and vehicles [51].

The primary purpose of ITSs is to avoid traffic congestion, which can be realized by providing real-time traffic information and detour routes. This is most closely related to traffic management optimization.

## 7. Discussion

### 7.1. Computer Vision-Based Pedestrian and License Plate Recognition

In the paper, we use a basic HOG algorithm for pedestrian recognition and a modified Kim’s algorithm for license plate recognition. To apply the methods for minimizing the computational load, we implemented the smart vehicular camera using the open source library (OpenCV), for convenient implementation, and conducted a performance evaluation. Many researchers have studied computer vision-based algorithms for pedestrian and license plate recognition. Those algorithms featured a trade-off between processing speed and accuracy. Therefore, we will undertake future work to improve processing speed and recognition accuracy by applying various algorithms.

### 7.2. Recognition Accuracy Based on Weather Conditions

The proposed smart vehicular camera used the image recognition library powered by OpenCV. The library has a training data set (INRIA) to operate only in fine weather conditions (sunny days). Thus, the recognition results can vary depending on weather conditions. To solve this problem, we use the training data in a variety of environments or the contextual training data for each situation. To use the contextual training data, the device requires a method to recognize weather conditions by a means other than the computer vision algorithm. Therefore, many studies are required to implement the available system in a variety of environments.

### 7.3. Optimal Value of N Frames for D-ROI Method

The proposed D-ROI method has a trade-off between processing speed and accuracy. When the number of D-ROI processing frames is increased, the recognition accuracy decreases, even though the processing speed of the recognition algorithm is high. We select the optimal value of *n* by considering the number of input images and the FPS value according to the image size.

### 7.4. Precision of Traffic Congestion Estimation

The actual and predicted traffic conditions may be different based on many variables. The proposed method estimates traffic conditions by recognized information, such as vehicle-to-vehicle distance, vehicle speed, and number of neighboring vehicles. However, the actual and predicted conditions can vary significantly depending on the number of lanes, traffic signal status, construction, and occurrence of accidents on the road. When the proposed smart vehicular camera is used in conjunction with such data, it would be possible to estimate better traffic conditions. These methods will be applied in future works.

## 8. Conclusions

A smart driver assistance system can provide a wide variety of status information using image processing and differs from the simple sensors of ADASs. Further, ADASs use the information obtained by various sensors attached to the vehicle and pass this information through a network in order to offer information to the driver or for vehicle control. A real-time traffic information system can estimate the traffic information for a road using the information collected and help the driver to drive safely on that road. 

In this study, we designed and implemented a real-time traffic information system that uses a smart camera device with image processing and network capabilities for application in smart driver assistance systems. For image processing, we designed D-ROI and DFS techniques to solve hardware performance problems. We implemented a parallel-processing-based smart camera vehicular application for efficient operation of the smart vehicle camera device and evaluated its performance. In addition, we proposed a traffic estimation model for providing the driver with real-time road traffic information on the basis of the collected information.

We evaluated the image recognition performance and found that the proposed methods improved the processing time by 1.58 times compared to that of the S-ROI and D-ROI methods. We found that applying the D-ROI method improved the processing time by 2.01 times compared to that obtained by applying the DFS method. In addition, we found that the processing time improved by 1.71 times when the proposed application was implemented by applying parallel processing. Finally, we found that the processing time improved by 15.4 times when all the proposed methods were applied in the application. We confirmed that four image recognition methods can be executed in real time. Moreover, we found, experimentally, that there was little or no change in the recognition accuracy when the proposed methods were used. 

A performance evaluation of the proposed traffic congestion estimation model to provide real-time traffic information showed that the average error rate of the improved scoring functions was 2.11% under the congested road condition. This error rate was 50% smaller than that obtained when the simple scoring functions were used. Under the light traffic condition, we found that it was difficult to use the intervehicle distance information and that the proposed estimation model showed an average error of 8.50%. Moreover, we found that the average error rate of the proposed model was 5.3%.
